# Resveratrol Activates Antioxidant Protective Mechanisms in Cellular Models of Alzheimer’s Disease Inflammation

**DOI:** 10.3390/antiox13020177

**Published:** 2024-01-31

**Authors:** Clara Bartra, Yi Yuan, Kristijan Vuraić, Haydeé Valdés-Quiroz, Pau Garcia-Baucells, Mark Slevin, Ylenia Pastorello, Cristina Suñol, Coral Sanfeliu

**Affiliations:** 1 Institut d’Investigacions Biomèdiques de Barcelona (IIBB), CSIC, 08036 Barcelona, Spain; clara.bartra@iibb.csic.es (C.B.); yuan_yi07@126.com (Y.Y.); kvuraic@stud.biol.pmf.hr (K.V.); ayd.valdes@gmail.com (H.V.-Q.); paugarbau@gmail.com (P.G.-B.); cristina.sunol@iibb.csic.es (C.S.); 2 Institut d’Investigacions Biomèdiques August Pi i Sunyer (DIBAPS), 08036 Barcelona, Spain; 3 PhD Program in Biotechnology, Facultat de Farmàcia i Ciències de l’Alimentació, Universitat de Barcelona, 08034 Barcelona, Spain; 4 School of Life Sciences, John Dalton Building, Manchester Metropolitan University, Manchester M15 6BH, UK; m.a.slevin@mmu.ac.uk; 5 Centru Avansat de Cercetari Medicale si Farmaceutice (CCAMF), Universitatea de Medicina, Farmacie, Stiinte si Tehnologie “George Emil Palade” din Targu Mures, 540142 Targu Mures, Romania; 6 Department of Anatomy and Embryology, Universitatea de Medicina, Farmacie, Stiinte si Tehnologie “George Emil Palade” din Targu Mures, 540142 Targu Mures, Romania; ylenia.pastorello@umfst.ro

**Keywords:** resveratrol, monomeric C-reactive protein, lipopolysaccharide, oxidative stress, inflammation, BV2, primary mixed glial cultures

## Abstract

Resveratrol is a natural phenolic compound with known benefits against neurodegeneration. We analyzed in vitro the protective mechanisms of resveratrol against the proinflammatory monomeric C-reactive protein (mCRP). mCRP increases the risk of AD after stroke and we previously demonstrated that intracerebral mCRP induces AD-like dementia in mice. Here, we used BV2 microglia treated with mCRP for 24 h in the presence or absence of resveratrol. Cells and conditioned media were collected for analysis. Lipopolysaccharide (LPS) has also been implicated in AD progression and so LPS was used as a resveratrol-sensitive reference agent. mCRP at the concentration of 50 µg/mL activated the nitric oxide pathway and the NLRP3 inflammasome pathway. Furthermore, mCRP induced cyclooxygenase-2 and the release of proinflammatory cytokines. Resveratrol effectively inhibited these changes and increased the expression of the antioxidant enzyme genes *Cat* and *Sod2*. As central mechanisms of defense, resveratrol activated the hub genes *Sirt1* and *Nfe2l2* and inhibited the nuclear translocation of the signal transducer NF-ĸB. Proinflammatory changes induced by mCRP in primary mixed glial cultures were also protected by resveratrol. This work provides a mechanistic insight into the protective benefits of resveratrol in preventing the risk of AD induced by proinflammatory agents.

## 1. Introduction

Resveratrol is a phenolic compound of the stilbene family. Its active *trans* form (*trans*-3,5,4’-trihydroxystilbene) has multiple health benefits shown in years of preclinical research. It is a powerful antioxidant that also exhibits anti-inflammatory, anti-aging and anti-neurodegenerative properties [1,2,3,4], among others. Animal studies showed that *trans*-resveratrol is able to cross the blood–brain barrier [5]. This supports the possibility that resveratrol could be protective for brain cells. Resveratrol is synthesized by more than 70 species of plants to stimulate cell defense in response to stress conditions. It is present in considerable amount in mulberries, lingonberries, cranberries, peanuts, the skin of red grapes, and in some medicinal herbs. In addition, resveratrol was initially associated with red wine as its main source. This misleading perception might lead to resveratrol being discredited after new studies showed no significant reductions in all-cause mortality with moderate drinking [6] and for concerns about possible alcohol addiction. However, moderate drinking in old men and women is consistently associated with a lower risk of dementia when compared with lifetime abstainers [7]. Whatever the active ingredient is in wine, resveratrol should be studied as a pure nutraceutical-type compound. In fact, therapeutic doses cannot be obtained from diet and require supplementation. Resveratrol doses in clinical trials with positive outcome were in the range of 150–250 mg/d for lowering blood pressure [8], for cerebrovascular and cognitive benefits in postmenopausal women [9,10], and cognitive benefits in overweight old men [11]. These authors hypothesized that the benefits found are at least partially based on the ability of resveratrol to modulate cerebral blood flow and vessel responsiveness during demand [9,10,11], and in association with improvement of the metabolic profile [8,11]. The proposed underlying molecular mechanisms include modulation of endothelial nitric oxide, decrease in oxidative stress, and activation of calorie restriction-like pathways (i.e., sirtuin 1 (SIRT1), AMP-activated protein kinase (AMPK) and nuclear factor-like 2). However, a number of clinical trials did not report benefits and thus, the results of meta-analysis are controversial [12]. We can speculate that either higher dosages or combination with other compounds to improve bioavailability could have worsened the outcome. Resveratrol is a hormetic agent and is not devoid of adverse effects at elevated concentrations [13,14]. Noticeably, a recent review of the limited number of clinical trials performed in Alzheimer’s disease (AD) patients concluded a delay of cognitive impairment following resveratrol supplementation in comparison with placebo [15]. However, the bioavailability and pharmacokinetics of resveratrol after oral administration are poor. Circulating resveratrol is mainly bound to albumin [16,17]. The hydrophobic structure of resveratrol may limit its absorption into target tissues. A main drawback is its rapid metabolism to less active glucuronide and sulfate conjugates [5,18,19]. The resulting low plasma and brain levels of *trans*-resveratrol after oral doses [5] have raised uncertainty about the administration regimen. Experimental strategies with innovative nanocarriers to improve the aqueous solubility and bioavailability of resveratrol have already shown benefits in the field of cancer therapeutics [20] and in brain delivery [21]. Research on innovative carriers and formulations of resveratrol to improve its bioavailability in humans is ongoing [22,23,24]. Improvements in pharmacokinetics will overcome the safety concerns in resveratrol dosing [25].

There is an interplay between oxidative stress and inflammation, as one may promote the other leading to a damaging spiral. With advancing age, an imbalance between the generation of reactive oxygen species (ROS) and its clearance leads to chronic inflammation. In turn, sustained inflammatory processes contribute to an increased risk of age-related diseases, including AD [26,27]. Resveratrol is effective in reducing oxidative stress in AD in vitro neuronal models [28,29,30,31,32]. These studies show that amyloid ß peptides induce oxidative stress in PC12 cells [28,29], human neural stem cells [30], HT22 cells [31] and primary neurons [32] that parallels cytotoxicity. The antioxidant protective mechanisms of resveratrol in these models included an increase in mitochondrial enzyme manganese superoxide dismutase (SOD2), inhibition of the redox-regulated transcription factor NF-kB, and activation of the master regulator of cell energy AMPK. Furthermore, resveratrol is effective against oxidative stress in immortalized lymphocytes from AD patients by increasing expression of genes encoding antioxidant enzymes and survival factors [33]. Resveratrol also shows antioxidant properties in in vivo rodent models of AD [31,32,34,35,36]. Transgenic mouse models of AD [31,32] and rat chemical AD models induced by colchicine [34,36] or angiotensin II [35] show oxidative stress as a deleterious mechanism associated with cognitive loss. Chronic intake of resveratrol inhibits oxidative damage by activating antioxidant systems in the brain, including SOD2 and reduced glutathione. Furthermore, as a phenolic agent it can also induce direct antioxidant effects.

Resveratrol has anti-inflammatory capacity in models of neurodegenerative disease, as shown by its downregulation of tumor necrosis factor α (TNFα) and other proinflammatory molecules [4,37,38,39]. Microglia are the key cells in the inflammatory processes associated with AD and in the interplay with oxidative stress and inflammation [40]. Previous studies showed that resveratrol inhibited amyloid ß-induced phenotype activation in microglial cell lines BV2 and N9 [41,42]. Several studies also showed that resveratrol protected against the proinflammatory effects of lipopolysaccharide (LPS) in microglial cells, using an in vitro model of neuroinflammatory diseases [43,44,45,46,47,48,49,50]. However, protective mechanisms of resveratrol that dampen microglial over-reactivity during AD progression are poorly characterized.

Here, we aimed to analyze the antioxidant protection mechanisms of resveratrol in BV2 microglia activated by monomeric C-reactive protein (mCRP) as a novel model of AD. The monomeric form of CRP is produced by activation and further disaggregation of the circulant pentameric CRP and has strong proinflammatory properties [51]. mCRP expression is strongly associated with the risk and progression of diseases dependent upon chronic inflammation [52,53,54]. In the brain it may increase AD risk after stroke [55,56]. In vivo and in vitro preclinical studies showed that mCRP induced the two main AD pathological markers, amyloid ß and hyperphosphorylated tau, and AD-like dementia in mice [56,57,58]. Resveratrol intake is known to decrease peripheral blood CRP levels in humans [59,60]. However, to our knowledge, the effects of resveratrol on mCRP within brain cells has not been studied to date. In addition, we used LPS as a reference agent involved in AD and other neurodegenerative diseases [61]. The proinflammatory effects of LPS on microglial cells are inhibited by resveratrol, as indicated above, which we analyzed further here. Resveratrol is effective against phenotype activation by LPS in both microglial cell lines and primary cultures. Therefore, we also aimed to confirm resveratrol inhibition of BV2 activation by mCRP in microglia grown in primary mixed glial cultures.

## 2. Materials and Methods

### 2.1. Cell Culture

BV2 microglial cells were used in this study (#ATL03001, ICLC, Banca Biologica e Cell Factory, Genova, Italy). The BV2 cell line was established from C57BL/6 mouse microglia, and it is a valued microglial model for the study of brain neuroinflammatory mechanisms [62]. BV2 cells were grown in RPMI 1640 medium (Biowest, Riverside, Newry and Mourne, UK) supplemented with L-glutamine 2 mM, gentamycin 50 µg/mL and 10% heat-inactivated fetal bovine serum (FBS) (all supplements were from Gibco, Thermo Fisher Scientific, Waltham, MA, USA). Cells were seeded in T25 flasks (NuncTM, Thermo Fisher Scientific) and sub-cultured 1:10 when they reached 80–90% of confluence.

Primary glial cultures were used for selected experiments to confirm the benefits of resveratrol against glia activation by mCRP. For these purpose, mixed glial cultures were prepared from cerebral cortices of C57BL/6 mice at 2–4 days of age following established procedures [63]. Briefly, cortices were minced and disaggregated into a single cell solution by enzymatic and mechanical processing. Cells were resuspended in DMEM:F12 with HEPES and L-glutamine (#31330038, Gibco). This medium was supplemented with gentamycin 50 µg/mL and 10% FBS (all culture reagents were from Gibco, Thermo Fisher Scientific). Cells were seeded in 96- or 24-well plates (Nunc, ThermoFisher Scientific, Munich, Germany) at 8 × 10^4^ cells/cm^2^ or in 8-well chamber slides (Lab-Tek Chamber Slides; Nunc, ThermoFisher Scientific) at 6 × 10^4^ cells/cm^2^. The medium was replaced every 5–7 days. Cultures contained mainly astrocytes and microglia.

### 2.2. Drugs and Experimental Design

mCRP was generated from the native CRP protein (YO Proteins, Ronninge, Sweden). A pure solution of CRP monomers was obtained by urea/EDTA chelation and subsequent dialysis, as previously described [57].

The LPS strain was E. coli 026:B26 (L-2654, batch #120M4028, Sigma-Aldrich, St. Louis, MO, USA). All other reagents were also from Sigma-Aldrich where not otherwise indicated.

For experiments, BV2 cells were seeded in 96- or 12-well plates (Nunc, ThermoFisher Scientific) at 5 × 10^4^ cells/cm^2^ or in 8-well chamber slides (Lab-Tek Chamber Slides; Nunc, Thermo Fisher Scientific) at 1 × 10^4^ cells/cm^2^. After 24 h, the medium was replaced with fresh culture medium without FBS, containing vehicle (DMSO) or resveratrol (1 µM to 50 µM, dissolved in DMSO). The final concentration of DMSO in all wells was 0.1%. After 1 h of pretreatment, cells were stimulated with the proinflammatory agents mCRP (50 µg/mL) or LPS (0.1 µg/mL, 1 µg/mL) and incubated for 24 h. Each treatment was performed in 2–3 wells and the whole experiment was repeated 3–5 times in cultures of different cell passage.

Primary glial cultures in well plates and chamber slides were used at 18–21 days in vitro. Treatments with resveratrol (10 µM to 50 µM) and mCRP (50 µg/mL) were performed following the same procedure used in BV2 cells. However, primary cultures were not subjected to serum starvation to avoid astrocyte damage. Each treatment was performed in 3–4 wells and the whole experiment was repeated in two independent primary cultures.

### 2.3. Nitrites Assay

Nitric oxide generation was determined with the colorimetric Griess reaction that detects nitrite (NO_2_^−^), a stable reaction product of nitric oxide and molecular oxygen, following standard procedures [57]. Nitrite levels in the fresh conditioned media were calculated with a nitrite curve in each 96-well plate. The results were expressed as a percentage of mCRP 50 µg/mL or LPS 1 µg/mL values, as peak values.

### 2.4. Enzyme-Linked Immunosorbent Assay (ELISA)

Conditioned media were collected and preserved at −70 °C until analysis. Levels of TNFα and interleukin 1ß (IL1ß) were measured using the Mouse TNF alpha uncoated ELISA kit and IL-1 beta Mouse Uncoated ELISA Kit, respectively (Invitrogen, Thermo Fisher Scientific). Samples were analyzed in duplicate. TNFα results were obtained in ng/mL and IL1β results in pg/mL.

### 2.5. Western Blotting

Protein extracts were obtained from the treated BV2 cells in 12-well plates at termination. Cultures were washed with cold PBS, homogenized in a cold RIPA buffer supplemented with protease and phosphatase inhibitors, and centrifuged. The supernatant concentration of proteins was determined by the Bradford protein assay (Bio-Rad, Hercules, CA, USA). Western blotting analysis of specific proteins was performed following standard procedures. Briefly, 10 µg of denatured protein samples were separated by SDS-PAGE at 100 V. Electrophoresed proteins were blotted onto PVDF membranes at 200 mA for 90 min. The membranes were then incubated for 1 h with a blocking agent followed by overnight incubation with the primary antibody at 4 °C. The primary antibodies used were anti-inducible nitric oxide synthase (iNOS) (1:1000; #610421, BD Bioscience, BD Transduction Laboratories, Franklin Lakes, NJ, USA), anti-nucleotide-binding domain, leucine-rich–containing family, pyrin domain–containing-3 (NLRP3) (1:1000; #NBP2-12446SS, Novus Biologicals, Bio-Techne, Centennial, CO, USA), anti-actin (20-33) (1:10,000; #A5060, Sigma-Aldrich, St. Louis, MO, USA) and anti-ß-tubulin (1:10,000; #T4026; Sigma-Aldrich). Membranes were rinsed and incubated for 1 h with the secondary antibodies. The antibodies used were sheep-anti-mouse HRP conjugated (1:2000; #NA931, Amersham, General Electric, Boston, MA, USA) and donkey-anti-rabbit HRP conjugated (1:2000; #NA934, Amersham). The proteins were visualized by enhanced chemiluminescence detection in a Chemidoc™ Imaging System (Bio-Rad, Hercules, CA, USA). The densitometric analysis was performed using Image Lab software (v3.0.1, Bio-Rad). The protein levels were normalized using ß-tubulin. Samples from all treatments were included on each membrane.

### 2.6. Real-Time Quantitative Polymerase Chain Reaction (qPCR)

Extraction of RNA from BV2 cells was carried out using TRIsureTM reagent (Meridian Bioscience, Bioline, London, UK), following manufacturer’s instructions. Samples were checked for RNA concentration and quality using a ND-1000 spectrophotometer (NanoDrop Technologies, Wilmington, DE, USA). Reverse transcription from RNA to cDNA was performed using a High-Capacity cDNA Reverse Transcription Kit (Thermo Fischer Scientific, Waltham, MA, USA). Three hundred ng of RNA per sample were loaded in a thermal cycler (FlexCycler, Analytikjena, Jena, Germany). cDNA samples were stored at −20 °C until analysis.

Gene expression was analyzed by qPCR using TaqMan^®^ Fluorescein amidite (FAM)-labeled specific probes (Thermo Fisher Scientific) and a Quantimix Easy Probe kit (Biotools, Madrid, Spain). The reaction mix containing 6.75 ng of cDNA was loaded in a CFX96TM Real-Time System (Bio-Rad). The Taqman assay probes were *Actb* (Actin beta, #Mm02619580_g1), *Cat* (Catalase, #Mm00437992_m1), *Clec7a* (C-Type lectin domain containing 7A, Mm01183349_m1), *Cox2* (Cyclooxygenase 2, Mm00478374_m1), Il6 (Interleukin 6, #Mm00446191_m1), *Nfe2l2* (NFE2 like BZIP transcription factor 2, Mm00477784_m1), *Nos2* (Inducible nitric oxide synthase, Mm00440502_m1), *Sirt1* (Sirtuin (silent mating type information regulation 2 homolog) 1, #Mm00490758_m1), and *Sod2* (Superoxide dismutase 2, #Mm01313000_m1). Samples were analyzed in duplicate. Results were normalized to actin beta gene expression using the comparative cycle threshold method (ΔΔCT).

### 2.7. Immunofluorescence Assay

Cells in chamber slides were fixed with 4% paraformaldehyde for 30 min, permeabilized with 0.2% Triton X-100 for 8 min and incubated with a blocking solution for 1 h.

BV2 were then incubated overnight at 4 °C with anti-nuclear factor κ-light-chain-enhancer of activated B cells (NF-κB) antibody, p65 subunit, active subunit, clone 12H11 (1:200; #MAB3026, Chemicon, Merck, Darmstadt, Germany). Slides were washed and incubated with Alexa Fluor 488-conjugated secondary antibody (1:1000; #A-11001, Molecular Probes, Thermo Fischer Scientific). Slides were mounted with Fluoroshield with DAPI (Sigma-Aldrich, Merck). Imaging was performed with an Andor Dragonfly 200 Spinning Disk confocal (Oxford Instruments, Abingdon, Oxfordshire, UK). 405 nm and 488 nm diode lasers were used to visualize DAPI nuclear fluorescence and NF-κB p65 fluorescence, respectively. Images were taken every 0.5 µm at 40x. Cell images at the midline of the nucleus were used for each cell analysis. Nuclear NF-κB p65 fluorescence was analyzed using ImageJ [64].

Primary cultures were incubated for 1 h at room temperature with anti-glial fibrillary acidic protein (GFAP) antibody (1:500; #Z0334, Dako, Agilent, Santa Clara, CA, USA) to stain astrocytes. The secondary antibody was conjugated with Alexa Fluor 546 (1:1000; Thermo Fisher Scientific). Microglia were stained with lectin from *Bandeiraea simplicifolia* conjugated with fluorescein (1:400; #L2895, Sigma-Aldrich). Slides were mounted with Fluoroshield (Sigma-Aldrich, Merck). Microphotographs to visualize changes in cell morphology were taken on a Nikon E1000 microscope.

### 2.8. Statistical Analysis

Results are expressed as the mean ± SEM. Normal distribution was checked with the Shapiro–Wilk test. Data were analyzed by two-way ANOVA with the factors: resveratrol and either mCRP or LPS. Tukey’s multiple comparisons test was used for the post hoc analysis. Statistical outliers were identified by Grubbs’ test (α = 0.05). The results were considered significant with a value of *p* < 0.05. The software used were GraphPad Prism v6.01 (GraphPad Software, La Jolla, CA, USA) and IBM SPSS Statistics v23 (IBM Corp., Armonk, NY, USA).

## 3. Results

### 3.1. Resveratrol Inhibited TNFα Production Induced by mCRP and LPS

TNFα is a master proinflammatory cytokine whose production by activated microglia in the brain is induced by a variety of agents and conditions.

**Figure 1:**
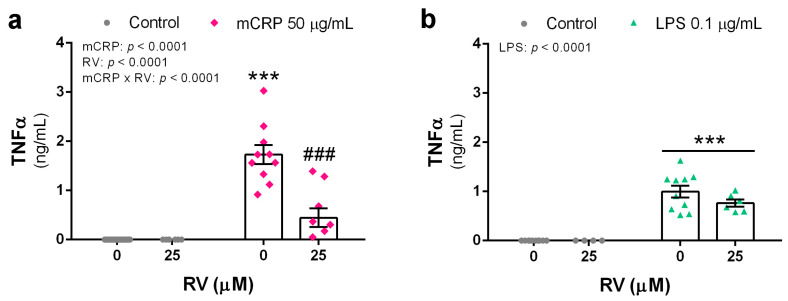
TNFα release by BV2 microglial cells activated with the proinflammatory agents mCRP (**a**) or LPS (**b**), in the absence or presence of resveratrol. Statistics: two-way ANOVA, significance of factors is indicated in graph; post hoc test, *** *p* < 0.001 compared to control, ### *p* < 0.001 compared to mCRP in the absence of resveratrol. RV, resveratrol.

mCRP at 50 µg/mL potently induced TNFα release by microglial BV2 cells which was inhibited by resveratrol to levels close to the control treatment (Figure 1a). LPS at 0.1 µg/mL induced less TNFα release than mCRP, while resveratrol showed a non-significant tendency to reduce the LPS effect at the tested concentration of 25 µM (Figure 1b).

Therefore, resveratrol protected against TNFα release from BV2 microglia after a proinflammatory injury, implying reduced activation of the inflammatory phenotype and subsequent downstream signaling.

### 3.2. Resveratrol Inhibited the Induction of the Nitric Oxide Pathway by mCRP and LPS

Nitric oxide is a signaling molecule produced by activated microglia via the enzyme iNOS. An excess of nitric oxide production will cause nitrosative and oxidative stress.

**Figure 2:**
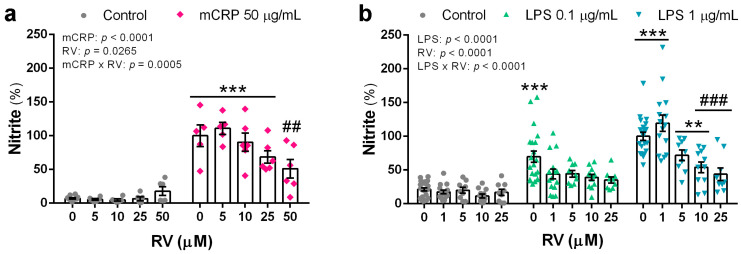
Nitrite levels in the culture media of BV2 microglial cells that indicate nitric oxide generation induced by mCRP (**a**) or LPS (**b**), in the absence or presence of resveratrol. Statistics: two-way ANOVA, significance of factors is indicated in graph; post hoc test, ** *p* < 0.01, *** *p* < 0.001 compared to control, ## *p* < 0.01, ### *p* < 0.001 compared to mCRP or LPS in the absence of resveratrol. Nitrite levels induced by LPS at 1 µg/mL without resveratrol or with resveratrol at 1 µM were higher than the corresponding treatments with LPS at 0.1 µg/mL (*p* < 0.05, not shown). RV, resveratrol.

mCRP at 50 µg/mL induced a significant increase in nitric oxide production, as detected by nitrite levels in the conditioned media, with a 14-fold increase over basal values. Resveratrol showed a concentration-response inhibitory effect that was statistically significant at the concentration of 50 µM (Figure 2a). LPS induced an approximate 3- and 5-fold increase in nitrite levels over basal levels at the concentration of 0.1 µg/mL and 1 µg/mL, respectively. Resveratrol reduced nitric oxide generation by LPS (Figure 2b).

**Figure 3:**
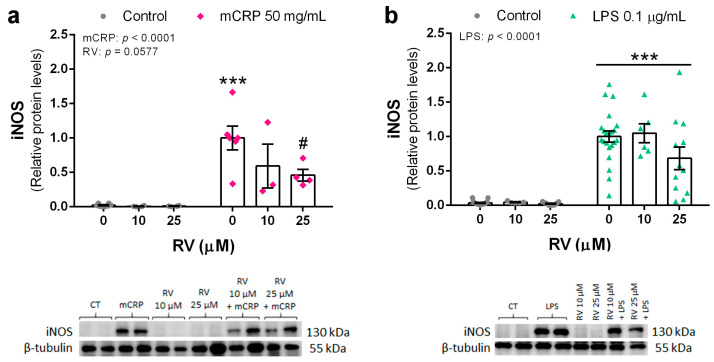
Protein levels of iNOS enzyme in BV2 microglial cells, induced by mCRP (**a**) or LPS (**b**), in the absence or presence of resveratrol. Statistics: two-way ANOVA, significance of factors is indicated in graph; post hoc test, *** *p* < 0.001 compared to control, # *p* < 0.05 compared to mCRP in the absence of resveratrol. Results and representative blots are displayed in the upper and lower panels, respectively. RV, resveratrol.

Protein levels of the iNOS enzyme were determined to confirm the protective action of resveratrol on the nitric oxide pathway. iNOS protein was significantly increased by mCRP at 50 µg/mL and by LPS at 0.1 µg/mL. Resveratrol efficiently inhibited the increase in iNOS levels by mCRP (Figure 3a). However, inhibition of LPS-induced iNOS by resveratrol up to 25 µM did not reach significance, probably due to the high dispersion of the data (Figure 3b).

Therefore, resveratrol was able to inhibit the induction of iNOS and subsequent production of NO, a main mechanism leading to oxidative stress and proinflammatory signaling by activated microglia.

### 3.3. Resveratrol Inhibited the Induction of the NLRP3 Inflammasome Pathway by mCRP and LPS

NLRP3 is a key sensor inflammasome found in microglia cells. This pathway is activated in response to diverse proinflammatory stimuli, including oxidative stress signals.

**Figure 4:**
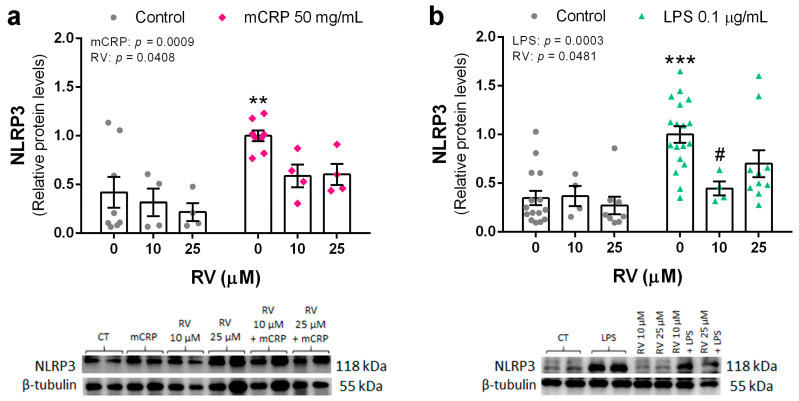
Protein levels of NLRP3 in BV2 microglial cells, induced by mCRP (**a**) or LPS (**b**), in the absence or presence of resveratrol. Statistics: two-way ANOVA, significance of factors is indicated in graph; post hoc test, ** *p* < 0.01, *** *p* < 0.001 compared to control, # *p* < 0.05 compared to LPS in the absence of resveratrol. Results and representative blots are displayed in the upper and lower panels, respectively. RV, resveratrol.

Protein levels of NLRP3 in BV2 cells were significantly increased by mCRP at 50 µg/mL and by LPS at 0.1 µg/mL. Co-incubation with resveratrol at 10 µM, or a higher concentration, reduced NLRP3 levels induced by both mCRP (Figure 4a) and LPS (Figure 4b).

These results showed that resveratrol was effective in avoiding the activation of the NLRP3 inflammasome pathway, which can lead to a spiral of neuroinflammation and further oxidative stress.

### 3.4. Resveratrol Inhibited the Activation of NF-ĸB by mCRP

Next, we studied the central mechanisms of mCRP as a much less characterized proinflammatory agent than LPS. First, we analyzed the nuclear translocation of NF-ĸB that initiates the transcription of genes involved in inflammation and oxidative stress, among other pathways.

**Figure 5:**
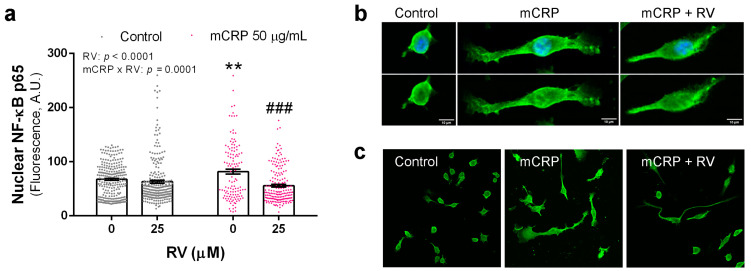
Immunofluorescence detection of NF-ĸB p65 in BV2 microglial cells. (**a**) Quantification of NF-ĸB p65 in the nucleus after exposure to mCRP, in the absence or presence of resveratrol. (**b**) Representative confocal images used for analysis. In the upper panel, images show green fluorescence for NF-ĸB p65 and blue fluorescence for DAPI nuclear staining; in the bottom panel, images of the same cells show green fluorescence. (**c**) Cell cultures stained for NF-ĸB p65 shown at lower magnification. Statistics in (**a**): two-way ANOVA, significance of factors is indicated in graph; post hoc test, ** *p* < 0.01 compared to control, ### *p* < 0.001 compared to mCRP in the absence of resveratrol. RV, resveratrol, A.U., arbitrary units.

Resveratrol decreased the nuclear content of p65 subunit of NF-ĸB after mCRP activation in BV2 microglial cells (Figure 5a). p65 and p50 form the most common heterodimers of the NF-ĸB complex, and increased detection of p65 in the cell nucleus indicates NF-ĸB activation. Morphological changes of polarization were observed in the mCRP treated microglia that were partially reverted by resveratrol. Confocal microscopy images of a representative cell from each treatment are shown in Figure 5b and images at lower magnification are shown in Figure 5c.

Therefore, the analysis of NF-ĸB showed that resveratrol protected against its activation and subsequent downstream deleterious mechanisms.

### 3.5. Resveratrol Inhibited the Induction of Proinflammatory Genes by mCRP

In a second step, we analyzed the changes in the expression of selected proinflammatory genes induced by mCRP and their modulation by co-incubation with resveratrol.

**Figure 6:**
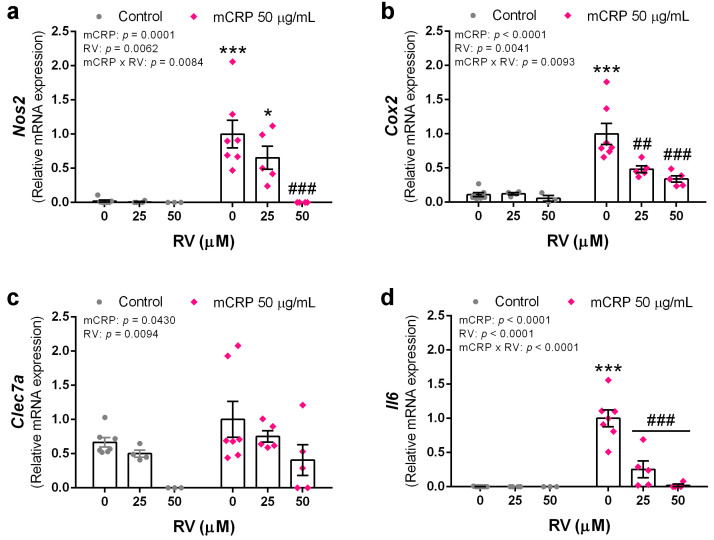
Expression levels of proinflammatory genes in BV2 microglial cells, induced by mCRP in the absence or presence of resveratrol. (**a**) *Nos2*. (**b**) *Cox2*. (**c**) *Clec7a*. (**d**) *Il6*. Statistics: two-way ANOVA, significance of factors is indicated in graph; post hoc test, * *p* < 0.05, *** *p* < 0.001 compared to control, ## *p* < 0.01, ### *p* < 0.001 compared to mCRP in the absence of resveratrol. RV, resveratrol.

Increased expression of *Nos2*, the gene codifying for iNOS, in mCRP treated BV2 confirmed the activation of the nitric oxide pathway. Resveratrol inhibited iNOS production at the gene level (Figure 6a).

The cyclooxygenase pathway is also activated by mCRP, as shown by increased *Cox2* expression. Similarly to the nitric oxide pathway, resveratrol inhibited cyclooxygenase 2 production and prevented the proinflammatory effects of its enzymatic activity (Figure 6b).

mCRP increased the expression of *Clec7a*, a gene codifying for a pattern recognition receptor in microglia. Interestingly, resveratrol decreases *Clec7a* mRNA levels in mCRP treated BV cells and in control cells. Therefore, resveratrol decreased both, the basal levels and those induced by mCRP, showing a preventive and protective function against downstream inflammatory processes (Figure 6c).

Interleukin 6 is a first line cytokine in the brain and the expression of *Il6* gene was increased by mCRP and inhibited by resveratrol (Figure 6d).

Taken together, resveratrol showed powerful anti-inflammatory properties by inhibiting the transduction of genes that trigger a variety of pathways.

### 3.6. Resveratrol Induced Antioxidant Genes to Protect against mCRP

Finally, we analyzed the changes in the expression of antioxidant and detoxifying genes induced by resveratrol that may protect against mCRP injury in a scenario of intertwined oxidative stress and inflammatory processes.

**Figure 7:**
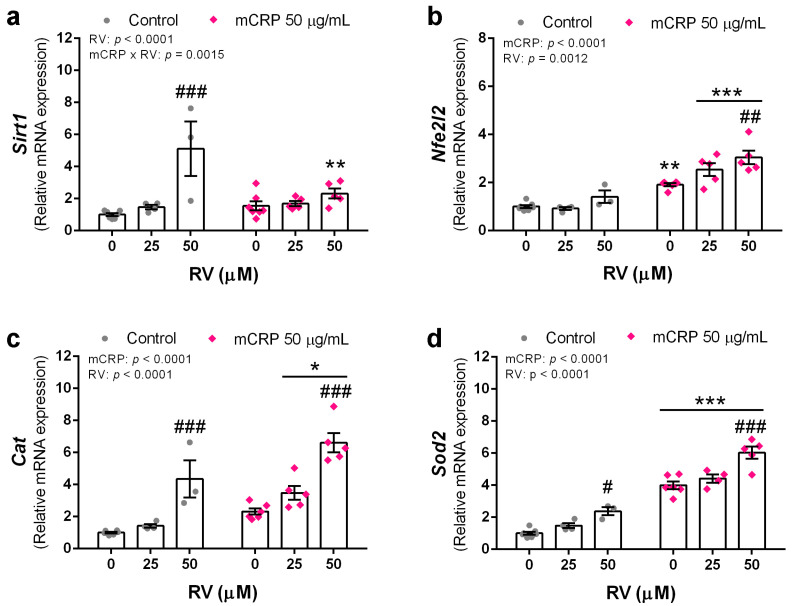
Expression levels of antioxidant genes induced by resveratrol in BV2 microglial cells, either control or mCRP treated. (**a**) *Sirt1*. (**b**) *Nfe2l2*. (**c**) *Cat*. (**d**) *Sod2*. Statistics: two-way ANOVA, significance of factors is indicated in graph; post hoc test, * *p* < 0.05, ** *p* < 0.01, *** *p* < 0.001 compared to control, # *p* < 0.05, ## *p* < 0.01, ### *p* < 0.001 compared to the absence of resveratrol. RV, resveratrol.

Resveratrol increased *Sirt1* gene expression as expected, although the effect was lower in the presence of mCRP (Figure 7a). The encoded protein SIRT1 can upregulate antioxidant and anti-inflammatory genes.

Resveratrol induced the expression of *Nfe2l2*, but in this gene the effect was mainly in the cells treated with mCRP. mCRP itself also induced this transducer of antioxidant and detoxifying genes (Figure 7b).

As probe of their protective antioxidant mechanisms, resveratrol induced the expression of the two first line defense genes *Cat* and *Sod2*. mCRP also induced an increase in the expression of both genes (Figure 7c,d, respectively). Catalase is widely present in the cytoplasm and superoxide dismutase 2 in the mitochondria.

Therefore, resveratrol induced powerful antioxidant and detoxifying mechanisms.

### 3.7. Resveratrol Inhibited Proinflammatory Changes Induced by mCRP in Mixed Glial Cultures

We used primary mixed glial cultures to test the inhibitory effect of resveratrol on microglia activation by mCRP in a physiological setting.

**Figure 8:**
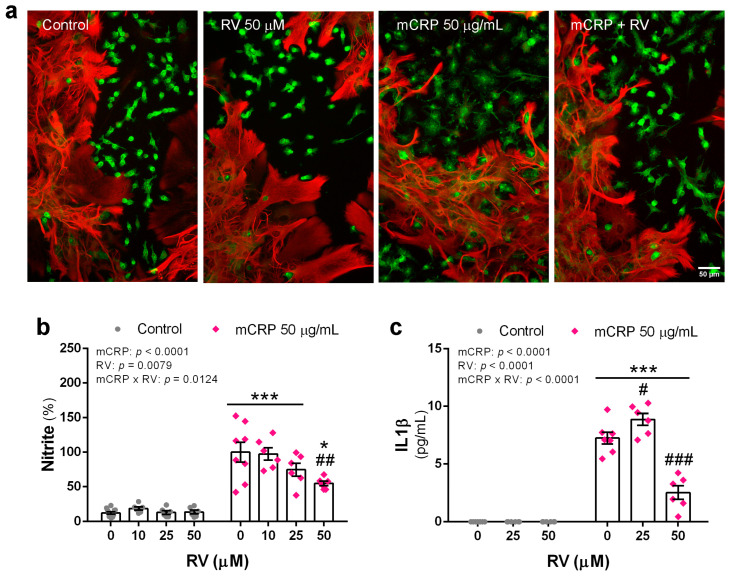
Proinflammatory changes induced by mCRP in primary mixed glial cultures in the presence or absence of resveratrol. (**a**) Microphotographs of cultures showing astrocytes (GFAP, red fluorescence) and microglia (lectin, green fluorescence) submitted to treatments as indicated in the figure. (**b**) Nitrite levels in the culture media indicative of nitric oxide generation. (**c**) IL1ß release to the culture media. Statistics: two-way ANOVA, significance of factors is indicated in graph; post hoc test, * *p* < 0.05, *** *p* < 0.001 compared to control, # *p* < 0.05, ## *p* < 0.01, ### *p <* 0.001 compared to mCRP in the absence of resveratrol. RV, resveratrol.

Images of the cultures did not evidence significant morphological changes of astrocytes with mCRP treatment. However, the microglia changed to a larger cell with flat morphology in the presence of mCRP. These changes were greatly reduced by resveratrol. Representative images of glial cultures are shown in Figure 8a.

Analysis of nitrite levels in the conditioned media showed an increase in nitric oxide release by cultures exposed to mCRP that was partially inhibited by resveratrol in a concentration-response effect (Figure 8b).

Similarly, resveratrol significantly inhibited the mCRP-induced increase in the release of the key cytokine IL1ß (Figure 8c).

## 4. Discussion

Resveratrol has been shown to protect against many harmful agents and conditions, but it had not previously been tested against mCRP. The protective results of resveratrol against mCRP shown here are relevant for possible future therapies in diseases involving this activated harmful form of CRP. Furthermore, the mCRP pathways uncovered here are relevant for the control of neuroinflammatory diseases where it may have a role.

In this study, we showed the protective action of resveratrol in two in vitro models of BV2 cells, treated with either LPS or mCRP, which mimic the aberrant activation of microglia associated with AD and other neurodegenerative diseases. LPS is a used in vivo model of AD neuroinflammation [61,65]. The LPS recognizing receptor, Toll-like receptor 4 (TLR4), is mainly expressed on microglia. In addition, TLR4 is also activated by amyloid ß, thus sharing downstream pathways with LPS [66]. Therefore, LPS-activated microglial cultures are a reliable model to study AD neuroinflammation in an in vitro setting. BV2 microglial cells have been used extensively to characterize the mechanisms of protective agents against LPS. mCRP is a novel and promising agent in the study of proinflammatory mechanisms associated with the onset and progression of AD. In a previous study, we had shown protection of mCRP dementia in the mouse by the anti-inflammatory agent N-[1-(1-Oxopropyl)-4-piperidinyl]-N’-[4-(trifluoromethoxy)phenyl]urea (TPPU) [57]. TPPU is an inhibitor of the soluble epoxide hydrolase enzyme and therefore enhances the concentration of the beneficial epoxyeicosatrienoic acids [67]. Furthermore, we showed that mCRP at the concentration of 100 µg/mL activated the nitric oxide pathway in BV2 microglia which was inhibited by TPPU [57]. Here, we used a lower concentration of 50 µg/mL mCRP and analyzed several inflammatory pathways known to be induced by LPS. Unlike LPS, the sequence of mCRP on microglial signaling is not known. mCRP may interact with the cell through a cholesterol binding sequence (a.a. 35–47) [68] that allows its attachment to the membrane lipids, complement component C1q and other elements [68,69]. However, we found that mCRP induces BV2 polarization to an activated phenotype as does LPS.

Resveratrol inhibited the aberrant activation of the nitric oxide pathway and the NLRP3 inflammasome pathway in both proinflammatory models, mCRP and LPS. The nitric oxide/NOS system plays an important role in many physiological processes. From the different NOS isoforms, iNOS is expressed by activated microglia. *Nos2* is the gene that codifies for the inducible form. This gaseous molecule modulates inflammatory cascades and therefore it may cause neuroinflammation [70]. In addition, excess production of nitric oxide together with insufficient antioxidant defense contribute to the unbalanced redox state associated with AD and other neurological diseases [71,72]. Furthermore, the multiprotein oligomer NLRP3 belongs to the inflammasome family and is a crucial player on the innate immune response. However, its aberrant activation may cause inflammatory damage and contribute to the progression of neurodegenerative diseases, such as AD [73,74]. A third pathway involved in neuroinflammatory processes is the production of prostaglandins and other downstream arachidonic acid metabolites through increased cyclooxigenase activity. From the two enzyme isoforms, COX-1 and COX-2, the latter is more likely involved in neurodegenerative processes due to its higher expression in brain [75,76]. The relevance of this pathway in neurodegeneration is more controversial than that of nitric oxide and the NLRP3 inflammasome. However, resveratrol also counteracted this pathway, as shown by inhibition of *Cox2* transduction.

Remarkably, resveratrol showed powerful protective properties against mCRP by inhibiting the three major signaling pathways discussed above that lead to downstream cascades of proinflammatory and pro-oxidant mediators. For instance, resveratrol counteracted the increase in TNFα cytokine levels and IL6 cytokine gene expression by mCRP. TNFα is involved in innate immunity and inflammation signaling and can cause necrosis or apoptosis. Genetic or pharmacological inhibition of TNFα was proposed to prevent or decrease the progression of AD pathology [77,78]. IL6 can promote a homeostatic or pathological role in the brain depending on the stimulus [79]. IL6 levels in the brain in a setting of neuroinflammation is a proposed target in the fight against AD [80].

Resveratrol protective effects shown here against LPS confirmed previous reports in BV2 microglia challenged with this proinflammatory agent [44,45,46,81].

We also used primary mixed glial cultures to confirm the resveratrol protection against mCRP as a novel agent in study. We showed a response pattern to mCRP and resveratrol similar to that of BV2 cells. We found a protective response against activation of the iNOS pathway. Furthermore, resveratrol inhibited the increased generation of IL1ß. One pathway inducing this cytokine is the NLRP3 inflammasome [82]. These results confirm that the main target cells of proinflammatory agents are microglia, as shown for LPS in a comparison between mixed cultures and pure microglia cultures [83]. Microglia are the main innate immune cells in the brain [84]. However, astrocytes contribute to the innate immune response. These cells can also release cytokines and other proinflammatory mediators, especially under conditions of sustained neuroinflammation such as in the AD brain [85]. It should be noted that resveratrol was able to normalize the levels of the three main pro-inflammatory cytokines IL1ß, IL6 and TNFα in either primary cultures or BV2 cells.

In the analysis of early signaling mechanisms, we found here that resveratrol increased *Sirt1* expression in the BV2 microglia, which would lead to an increase in the synthesis of the protein SIRT1. This is consistent with the known feature of resveratrol as an activator of SIRT1 [86]. SIRT1 is a deacetylase enzyme that regulates the activity of several transcriptional factors and enzymes involved in cell metabolism, stress defense and survival [87,88]. It may be coupled to another nutrient sensing molecule, AMPK [89]. SIRT1 pathway is considered the main effector of resveratrol benefits in experimental models of AD [90], although this pleiotropic agent can act through several mechanisms [4]. We previously showed an increase in Sirt1 expression parallel to the antioxidant and anti-aging effects in lymphocytes from AD patients treated with resveratrol [33]. We also showed that *Sirt1* overexpression induced cognitive improvement in transgenic AD mice [91] similar to that of resveratrol supplementation [92]. Here, we propose that SIRT1 is the main mediator of antioxidant and anti-inflammatory mechanisms leading to the protection against mCRP and LPS.

Studies reported in N9 microglial cells conclude that SIRT1/AMPK pathway is involved in the protective effect of resveratrol against the activation of the NLRP3 inflammasome and NF-kB by LPS [47]. In BV2, activation of SIRT1 by resveratrol and its inhibition with an antagonist demonstrated the involvement of this deacetylase in the modulation of proinflammatory cytokines induced by LPS [48]. In BV2 microglia challenged with mCRP, SIRT1 induced by resveratrol might modulate both key pathways NLRP3 and NF-κB leading to inhibition of downstream inflammatory targets. SIRT1 may also mediate the antioxidant gene response of resveratrol against mCRP oxidative damage [93]. SIRT1 is an activator of nuclear factor E2-related factor 2 (Nrf2), whose *Nfe2l2* gene expression was shown here to be activated by resveratrol and mCRP. Nrf2 transcriptional activity subsequently induced gene expression of antioxidant genes, as shown by parallel increases in *Cat* and *Sod2* expression in BV2 cells. Interestingly, hub transcription factors such as NF-ĸB and Nrf2 are stimulated by resveratrol, but also respond to ROS [94] as shown here for *Nfe2l2* gene expression, in a crosstalk between the mechanisms of this hormetic agent and those of mCRP.

**Figure 9:**
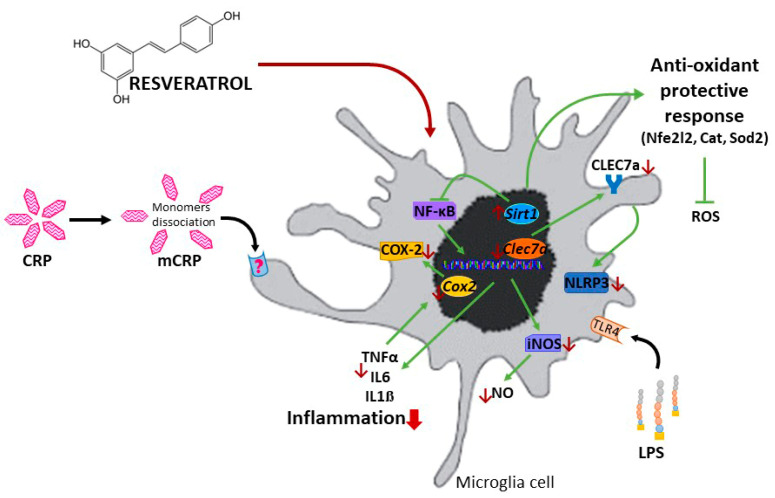
Schematic representation of the protective mechanisms of resveratrol against the proinflammatory agent mCRP and LPS.

A schematic representation of the proposed protective mechanisms induced by resveratrol against proinflammatory activation of microglial cells is displayed in Figure 9. Results for mCRP are those found in this study. Results for LPS are from this study and previously published data, as referred to above. We speculate that anti-inflammatory mechanisms of resveratrol against both agents, mCRP and LPS, are common.

We showed molecular targets of resveratrol in activated microglia cells, although pharmacodynamics was not further studied at the subcellular organelle level. Intracellular trafficking of resveratrol to cellular targets in these cells is also unknown. Other authors reported some advances in the analysis of the intracellular trafficking of resveratrol in peripheral cell types [17,95,96]. It is proposed that resveratrol crosses the cell membrane via passive diffusion, endocytosis via lipid rafts, or binding to receptors intro rafts [17,95]. Resveratrol may accumulate primarily in the endosomal−lysosomal system. It would then be released into the cytoplasm to perform its actions on key targets. Early endosomes, late endosomes, lysosome vesicles and other cell organelles can be identified with fluorescent labels [97]. Colocalization studies of these organelles with labeled resveratrol or resveratrol coupled to labeled nanocarriers [98,99] will show the time course and spatial distribution of resveratrol in cellular compartments. We wish to develop new studies using fluorescently tagged resveratrol to follow its entry into cultured cells or the mouse brain using confocal microcopy technologies. Specifically, spinning disk confocal microscopy and infrared laser multiphoton microscopy will be used for in vitro and in vivo studies, respectively. These studies would reveal subcellular targets in microglia and other brain cells. Despite its low bioavailability in vivo, resveratrol induces undoubted brain benefits, as demonstrated by a large number of preclinical and clinical studies for AD [4,15,100]. Promising results in enhancing stability, pharmacokinetics and pharmacodynamics of resveratrol in in vitro and in vivo cancer studies were recently critically reviewed [96]. Likewise, improved formulations of resveratrol to avoid its poor bioavailability and pharmacokinetics in brain tissue will improve its neuroprotective potential.

Therefore, the analysis of resveratrol protection in both AD models of inflammation, mCRP and LPS, strengthened the value of this nutriceutical agent. However, all the experimentation is carried out in in vitro models. We can speculate on the validity of the findings in humans, although further confirmation in in vivo preclinical models is required. Furthermore, there are other inflammatory/oxidative stress mechanisms not analyzed here that may play a role in the benefits of resveratrol on microglia. This is the case of the modulation of nicotinamide adenine dinucleotide phosphate oxidase 2 [101,102] and mitogen-activated protein kinases (ERK1/2, JNK, and p38) [45]. Recent insights into the contributions of resveratrol against persistent neuroinflammation include improving mitochondrial status and glucose metabolism in microglia [47,99,103].

## 5. Conclusions

Resveratrol protected against the polarization of BV2 microglia into an activated phenotype induced by two critical proinflammatory agents, LPS and mCRP.

The characterization of mCRP proinflammatory and pro-oxidant mechanisms in BV2 microglia showed the activation of the inflammatory/oxidative cascades of nitric oxide, NLRP3 inflammasome and COX-2 in this novel in vitro model.

Resveratrol protective mechanisms against mCRP required the modulation of SIRT1, Nrf2, and NF-ĸB pathways that reduced downstream inflammatory mediators and, most notably, induced antioxidant enzymes.

Resveratrol protective mechanisms against activation to proinflammatory phenotype by mCRP was confirmed in primary mixed glial cultures.

## Data Availability

Data supporting the conclusions of this article are contained in the corresponding figures; full raw data will available upon request without restrictions.
